# Coagulation and Flocculation before Primary Clarification as Efficient Solutions for Low-Density Microplastic Removal from Wastewater

**DOI:** 10.3390/ijerph192013013

**Published:** 2022-10-11

**Authors:** Piotr Jachimowicz, Agnieszka Cydzik-Kwiatkowska

**Affiliations:** Department of Environmental Biotechnology, Faculty of Geoengineering, University of Warmia and Mazury in Olsztyn, Słoneczna 45G, 10-709 Olsztyn, Poland

**Keywords:** microplastic, wastewater, wastewater treatment plant, coagulants, flocculants, micropollutants

## Abstract

Microplastic (MP) removal from wastewater was investigated using various types and doses of commercial coagulants (PIX, PAX) and flocculants (FPM, PEL, FCT) before primary clarification in a wastewater treatment plant (WWTP). Dosing with FPM, PIX, and PEL caused small MPs (180–212 µm) to be transferred mainly to the settled sludge (up to 86.4% of MP at a dose of 5 mL FMP/m^3^), while dosing of FCT and PAX caused these MPs to be transferred to the floated sludge (up to 64% MP at a dose of 5 mL PAX/m^3^). The efficiency of MP removal from wastewater was the highest (90%) with 2.5 mL PAX/m^3^; the generated primary sludge had a low MP content and could be safely managed in subsequent stages of sludge treatment. At the highest doses, PIX significantly increased the removal of P-PO_4_ (up to 94%) and COD (up to 73%). FPM and FCT resulted in over 40% efficiency of ammonium removal—such disturbance in wastewater composition may negatively affect further biological treatment. Effective removal of MP in the mechanical part of WWTP resulting from coagulation and flocculation enables the safe use of the excess sludge for agricultural purposes.

## 1. Introduction

Microplastic (MP) is a micropollutant commonly present in wastewater. Primary microplastics include industrial ‘scrubbers’ used to blast clean surfaces, plastic powders used in molding, and plastic nanoparticles used in various industrial processes. Kalčíková et al. [1] showed that concentrations of microbeads varied from 0.42 to 11.12 mg per 100 mL of the tested personal care products (facial and body scrubs). As primary MPs utilized in consumer products are washed down the drain, there is a growing concern regarding the direct emission of MP into aquatic environments [2]. Hidayaturrahman and Lee [3] have shown that microbeads comprise the highest percentage of microplastic particles (with regards to the shape) present in effluents the different wastewater treatment plants (WWTPs). Due to the smooth surface of microbeads, lower adsorption capacity in the mechanical part of WWTPs is expected compared to that of irregular shaped particles of MP [4].

The presence of MP in wastewater entering the biological part of WWTPs, with MP accumulation in the biomass, negatively affects the metabolic potential of the biomass. It was observed that polyether sulfone (PES) at a concentration of 0.5 g MP/L slightly inhibited ammonium removal and significantly decreased the specific rate of nitrate reduction. The presence of PES inhibited the activity of nitrite oxidase in biomass and increased the metabolism of amino acids. After increasing the level of nitrate reductase 1 in bacterial cells as a result of PES MP addition, the quantities of cytochrome c-containing subunit II and cytochrome b-containing subunit I decreased, leading to the accumulation of nitrite nitrogen and affecting nitrogen metabolism. High-throughput sequencing showed that the presence of PES in wastewater reduced the abundance of denitrifying *Bacillales* in aerobic granules and stimulated the growth of *Anaeroline,* which can anaerobically metabolize hydrocarbons that are present in MP [5].

The highest MP removal occurs in the mechanical part of wastewater treatment plants (WWTPs) (50–98%), and is related to the high efficiency of removing suspended solids with adsorbed MPs [6]. Most technological solutions for MP removal that currently in testing postulate the addition of a third treatment stage in the form of membranes, sand traps, coagulation, or ozonation chambers [7]. However, the addition of a third treatment stage increases the WWTP’s operating costs and does not prevent the accumulation of MPs in the sludge [3]. Research by Park et al. [8] at 50 South Korean wastewater treatment plants (WWTPs) showed that the concentration of MP in the influent varied from 14 to 470 MP/L. High MP removal efficiency in wastewater treatment systems, reaching > 90% [9,10], results from the fact that large amounts of MP (22–89%) are transferred to sludge. Koutnik et al. [11] reported that the concentration of MP in sewage sludge might reach 39020 MP/kg of dry mass (DM).

In wastewater treatment, coagulation lowers the electrokinetic potential of colloidal particles and suspensions and allows their aggregation. During coagulation, chemicals react with wastewater, and some soluble pollutants are precipitated and then removed by settling [12]. In the studies of El-Gohara et al. [13], the addition of aluminum coagulant (600 mg/L) to wastewater from the personal care-products industry resulted in efficiencies of COD and total suspended solids (TSS) removal of 76% and 94%, respectively. Coagulation efficiency depends on the appropriate selection of coagulant dose, and for dose optimization, the total costs of the process must be considered [14]. To remove TSS from wastewater, flocculants can be used that cross-link suspensions to form large, quickly settling agglomerates [15]. There is no data in the literature on using coagulants or flocculants (C/Fs) to intensify MP removal from wastewater in the primary clarifier.

The study investigated the effect of the dose and type of C/Fs on the efficiency of polyethylene (PE) MP removal from wastewater before the primary clarifier. It also aimed to determine how the amount and type of C/Fs affect the removal of other pollutants from wastewater in the context of disturbance of biological wastewater treatment. Removal of MPs in the mechanical part of WWTPs will eliminate the negative impact of MP on the biological part of the WWTPs, and will enable agricultural use of the excess sludge. In this study, MP in the form of PE was tested because this type of plastic predominates in wastewater that contains personal care products [16].

## 2. Materials and Methods

### 2.1. Substrate

Wastewater for the experiment was collected before the primary clarifier at the WWTP “Łyna” in Olsztyn (Poland). The concentration of pollutants in the wastewater averaged 436.0 ± 32.0 mg COD/L, 41.4 ± 5.3 mg N-NH_4_/L, 18.5 ± 2.5 mg P-PO_4_/L, 2740 ± 350 mg DM/L. The pH and salinity (mS/cm) of the wastewater were 7.81 and 0.98, respectively.

Known concentrations of fluorescent polyethylene microbeads (Figure S1; Cospheric, Santa Barbara, CA, USA) with diameters of 710–850 µm (green) and 180–212 µm (blue) were added to the wastewater.

### 2.2. Experiment Set-Up

The experiment was carried out in 1000 mL beakers in a coagulation apparatus with paddle stirrers (Flock Tester AL 46-6, Aqualytic, Dortmund, Germany). Wastewater and the appropriate C/Fs were added to the beakers. The contents of the beakers were intensively mixed for 2 min (250 RPM), then slowly mixed for 28 min (100 RPM), followed by 15 min of settling.

In the experiment, flocculants Flopam EM 840 MEB (FPM), Praestrol k 255 l (PEL), Flocculant F-290 (FCT), and coagulants PIX 113 (PIX), PAX 18 (PAX) (Table 1) were used. Working solutions of C/Fs were prepared 24 h before use by diluting 1 mL (liquid form) or 1 g (dry form) of the respective C/F in distilled water. The working solutions were dosed into the beakers at doses of 1.0, 2.5, and 5.0 mL/L. Experiments and analyses were carried out in triplicate for each type and dose of C/F.

**Figure 1:**
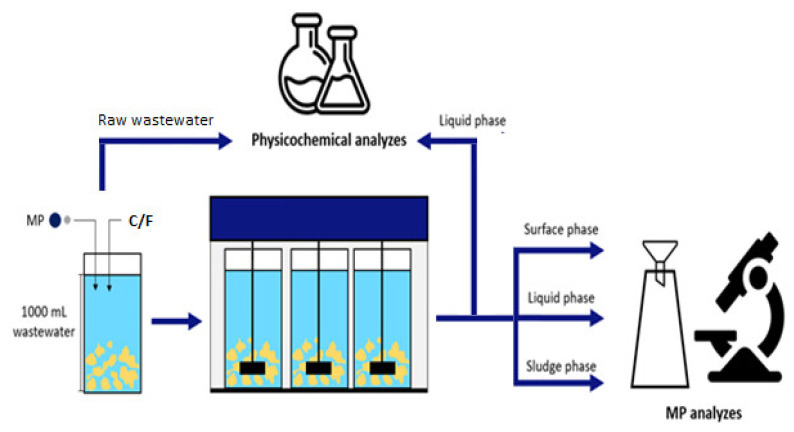
The scheme of the experiment.

Three fractions were collected from the experimental beakers: surface phase (upper 100 mL of wastewater and floated suspensions), liquid phase, and sludge phase (100 mL of sludge and wastewater from the bottom of the beaker); it was assumed that MP was present in the surface and sludge phase. Each collected fraction was filtered on filters with a pore diameter of 125 µm in a Büchner funnel at a constant vacuum of 500 g/cm^2^ (Figure 1). Each sample was filtered several times to avoid overloading a filter with suspended solids which may obscure the MP count. The readings from all filters were summed to describe the content of MP in a particular sample. Filtration was carried out in a laminar flow box to avoid loss of particles. The MP particles on the filters were counted using an optical binocular (Delta Optical SZ-450) with a 10× zoom in the darkroom, using UV to activate the fluorescent dye.

Chemical analyses of raw and liquid phases (in triplicate) included COD, TSS, N-NH_4_, and P-PO_4_ [17]. The pH was measured with a TitroLine easy (Donserv). Observations of the settled sludge were carried out using an optical microscope equipped with a camera (Nikon Eclipse 50I).

## 3. Results and Discussion

### 3.1. Removal of MP

The research assumed that the transition of MP from wastewater to floated or settled sludge would effectively eliminate MP in the primary clarifier, minimizing its negative effect on biomass in aeration tanks and on the quality of excess sludge. The studies by Petroody et al. [18] showed that the primary clarifier can remove 75.2% of the incoming MP and that the particle removal efficiency increased proportionally to the MP size. Plastic particles of 37–300 µm were removed with an efficiency of 70%, while particles larger than 500 µm were removed with an efficiency of 85%. Low-density MPs float on the surface of the wastewater with grease or oil and are mainly removed by surface skimming [19].

**Figure 2:**
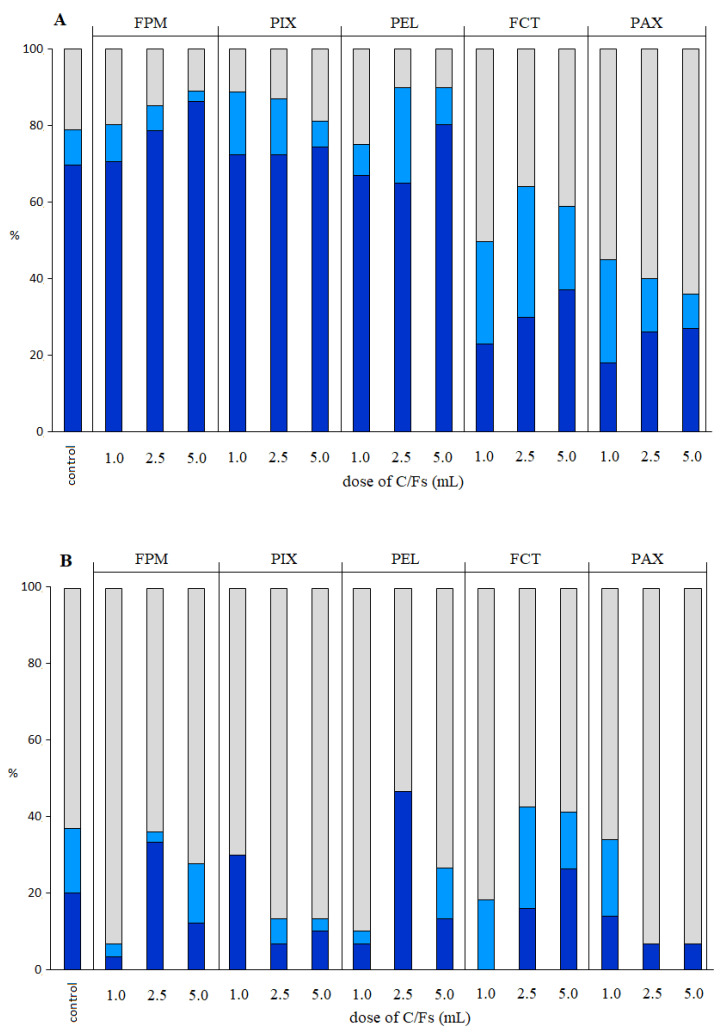
Distribution of MP with a diameter of 180–212 µm (**A**) and 710–850 µm (**B**) in the surface phase (grey), the liquid phase (light blue), and the sludge phase (dark blue).

In the present study, coagulants influenced the distribution of MP in the wastewater column (Figure 2A). Application of FPM, PIX, and PEL caused mainly smaller MP particles to be retained in the sludge phase (62.0–86.4%). In the cases of FCT and PAX, most of the MP was present in the surface phase (36.0–64.0%). For all C/Fs, except PEL, the percentage of MP particles in the liquid phase decreased with an increasing dose of C/Fs. Although different coagulants demonstrated different removal effects on MPs, their specific removal mechanism can still be explained by classical coagulation removal mechanisms, such as charge neutralization, adsorption, and sweep flocculation. The hydrolysates of metal coagulant can be adsorbed on the surfaces of negatively charged MPs, neutralizing the original charge on the MP surface and reducing the electrostatic repulsion, accordingly making the MPs unstable. The positively charged coagulant hydrolyzed monomers can adsorb the surrounding negatively charged MPs to form flocs [20]

In the controls, even without using C/Fs, the removal of MP was observed as a result of the sedimentation process. Researchers [1,21] reported that MP was captured in biomass of sludge settling at the bottom of the reactor. This was caused by the adhesion of dissolved organic matter particles to the MP structure, creating an eco-crown and decreasing hydrophobicity [22].

Most of the smaller MP particles (34.0% of all MPs) remained in the liquid phase and potentially entered the biological part of the WWTP when FCT was used (2.5 mL/L). The most efficient removal of small MPs from the liquid phase was observed at a dose of 5 mL FPM/L—only 4% of those MPs remained in the treated wastewater. Ma et al. [23] observed that dosing with C/F resulted in high efficiency of removal of MPs with small particle diameter. Addition to wastewater of 0.2 mmol/L of FeCl_3_·6H_2_O and 15 mg/L of cationic polyacrylamide flocculant led to 1–5% average efficiency of removal of MPs with diameters of 0.5–5 mm. In contrast, MP with diameters 140 µm and 15 µm were removed with 10% higher efficiency [24]. In the present study, MP in the form of granules was used. Granular MP is more complicated to remove than, e.g., MP in the form of fibers, due to the smaller reactive surface of granules, which limits the interaction with chemical compounds. Shahi et al. [25], in experiments on MP removal from water, reported that even at very high doses of coagulant, e.g., 50 mg/L (economically unjustified), the removal efficiency of granular MP did not exceed 32%. Our research shows that this efficiency was much higher (even up to 96%) when the coagulation process was applied to wastewater before the primary clarifier.

After the application of C/Fs, the larger MP particles (710–850 µm) were mainly found in the surface phase (53.3–93.4%) (Figure 2B). The percentage of larger MP particles in the liquid phase remained at the level of 2–10% for most of the C/Fs, only for 2.5 mL FCT/L did this percentage increase to 26.6%. Lofty et al. [26] reported that after a seven-day sampling period, a higher concentration of particles with 1000–5000 μm diameters was present in the scum than in the sludge (1271 vs. 771 MP/L) in the primary settling tank. The higher concentration of MPs in the scum resulted from a lower density than water, or from their flocculation with floating fats, oils, and grease. The tested MP was eliminated after applying 1.0 mL PIX/L, 2.5 mL PEL/L, and PAX at the two highest doses. The weight of MP particles with diameters of 710–850 µm was significantly greater than that of MP particles with diameters of 180–212 µm (0.321 mg vs. 0.007 mg). Therefore, the total weight of removed MP was determined by removing the larger MP particles. Murphy et al. [27] found that microbeads were effectively removed by skimming. This was consistent with other studies conducted by Michielssen et al. [28] and Sutton et al. [29]; both studies found that microbeads were absent in the effluent of WWTPs. In contrast, a survey conducted on WWTPs in New York showed that 4 out of 10 WWTPs still released microbeads [30]. This difference might be due to the different amounts of fat, grease, and oil in the wastewater, as these compounds are important for microplastics skimming.

### 3.2. Effectiveness of Pollutant Removal from Wastewater

**Figure 3:**
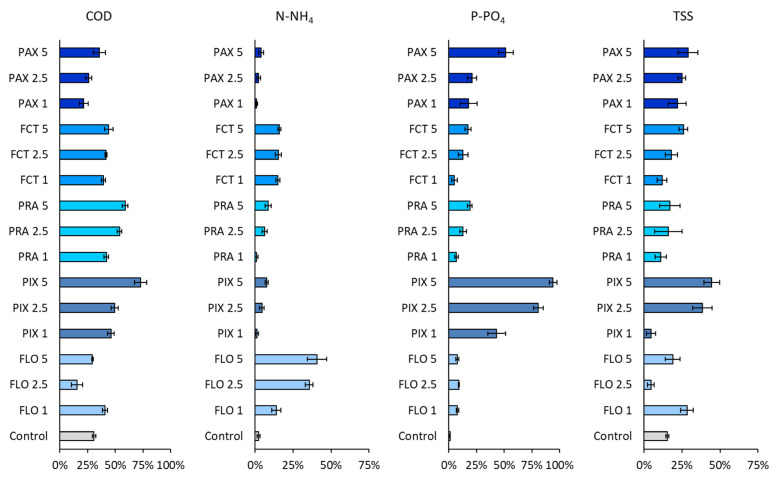
The effectiveness of pollutant removal from raw wastewater depending on the type and dose of C/Fs.

Based on the quality of wastewater after C/Fs treatment, a dendrogram was created, grouping samples with similar effectiveness of removal of individual pollutant indicators (Figure 3). Settling of wastewater for 15 min without the addition of C/Fs reduced the concentration of COD (31%) and total suspended solids (16%), but did not significantly affect the reduction of N-NH_4_ and P-PO_4_ (<2%). High efficiency of N-NH_4_ removal was observed for wastewater treated with FPM—36% and 41% of ammonium nitrogen was removed at doses of 2.5 and 5.0 mL/L, respectively. Such high efficiency of N-NH_4_ removal may result from the fact that FPM contains polyacrylamide, which can absorb N-NH_4_ on its surface [31].

Application of PIX at a dose ≥ 2.5 mL/L resulted in high efficiency of TSS removal (up to 45%). High efficiency of P-PO_4_ was observed for PIX and PAX. The efficiency of P-PO_4_ increased from 43% at the dose of 1 mL PIX/L, to 94% at the dose of 5 mL PIX/L. Fe(III) salts in PIX were dissociated, and the Fe(III) ions reacted with P-PO_4_—the obtained salts were precipitated. The efficiency of COD removal increased with increasing doses of C/Fs. The highest efficiency of COD removal was observed for PEL and PIX at 5 mL/L—the use of 5 mL PIX/L reduced the COD concentration in wastewater by over 70%. Efficient COD removal resulted from the combination of charge neutralization, entrapment, adsorption, and complexation with coagulant metal ions present in PIX into insoluble particulate aggregates [32]. Too much removal of one of the nutrients in the mechanical part of WWTP due to C/F application may affect the operation of the biological part of WWTP. COD is necessary for the effective biological removal of P and N. For example, lowering the COD/N ratio from 4.5 to 2.3 in the influent decreased denitrification efficiency and, consequently, the removal of nitrogen from wastewater [33]. Optimization of the dose of coagulants in the mechanical part of the WWTP is necessary to avoid the transfer of coagulants to the biological part. Philips et al. [34] showed that the continuous dosing of iron(III) salt to the activated sludge gradually inhibited total and nitrifying activity. This toxicity can be attributed to decreased pH due to forming iron hydroxides. The observed loss of nitrification was also partly a consequence of the negative effect of ferric iron on the structure of activated flocs.

**Figure 4:**
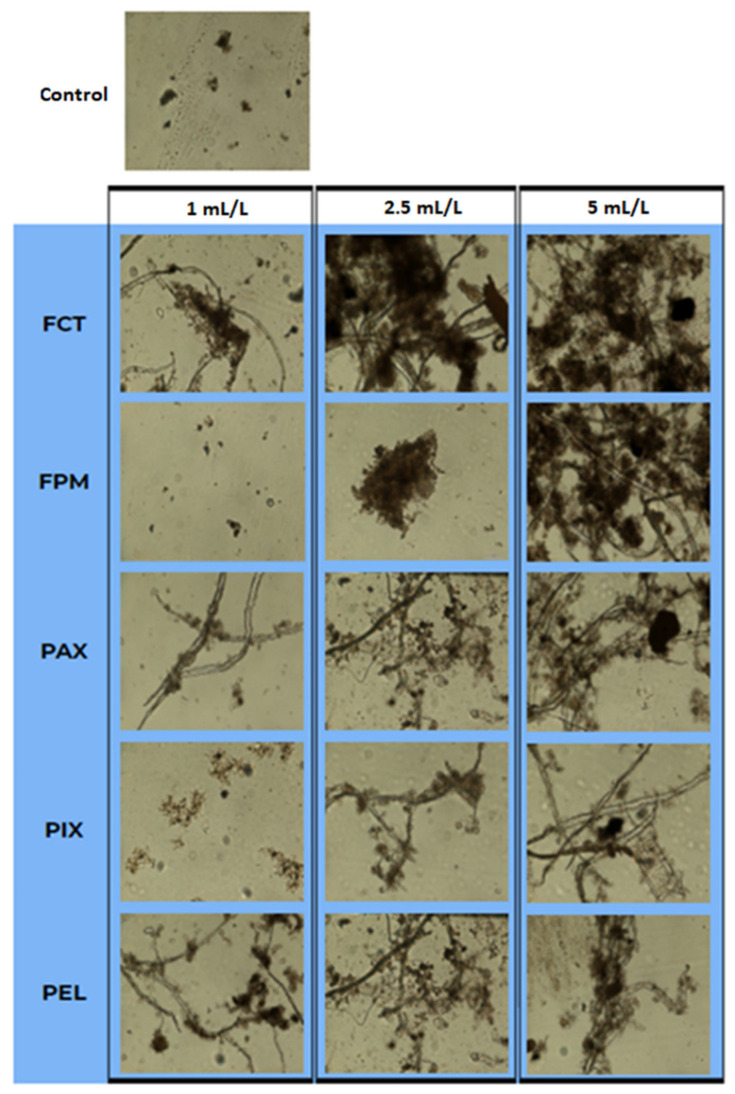
Microscopic analysis of solids from the sludge phase depending on the type and dose of C/Fs.

Microscopic analysis showed that the addition of C/Fs affected the structure of the settled agglomerates (Figure 4). In the control wastewater, suspensions with diameter <100 µm predominated. The largest suspensions with diameter > 1 mm were formed at the dose of 5 mL/L FCT and FPM. The presence of aromatic groups in FPM facilitates hydrophobic interactions between the flocculant polymer and the surface of the particle, which enhances agglomeration. Furthermore, the structure of this polymer, which is more branched than the other polymers, enables it to adsorb more particles and form denser aggregates [35]. In this study, microscopic analysis showed that increasing the C/F doses stimulated cross-linking and increased the density of aggregates. The high specific surface area of those aggregates favored the removal of undissolved pollutants from the liquid phase of the wastewater. Coagulants destabilize the surface charge of colloids and stabilize the surface charge of the suspended MPs, which allows the particles to come close enough to enable van der Waals interactions and particle agglomeration [36]. The maximum particle size that can be incorporated into an existing floc is proportional to the size of the floc [24]. The studies of Kalčíková et al. [1] showed that PE microgranules had a very high affinity for activated sludge and accumulated in sludge and fragmented organic matter at the bottom of the reactor.

**Figure 5:**
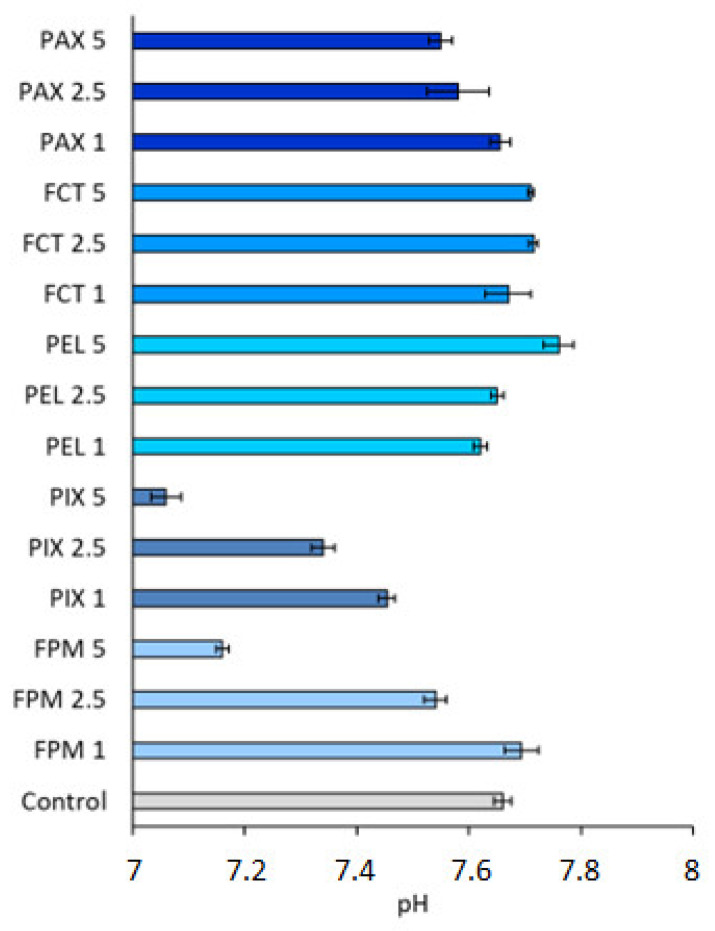
The pH of wastewater after dosing with C/Fs.

In all cases, the addition of C/Fs to wastewater changed the pH value (Figure 5). The change in pH value towards alkaline was noted for FCT and PEL. FPM and PIX decreased responses by maximum 0.50 and 0.54 pH units, respectively. The dose of 1.0 mL PAX/L increased the pH value; the other doses resulted in its decrease.

## 4. Conclusions

The addition of C/F to raw wastewater caused from 66.6 to 97.3% of MP to be transferred from wastewater to the floated or settled sludge, enabling effective MP removal in the primary clarifier. MPs with larger diameters (710–850 µm) were mainly found in the floated sludge. The highest efficiency of MP removal from wastewater (90%) was observed for 2.5 mL PAX/L—the low content of MP in the generated primary sludge enabled its safe use in subsequent stages of sludge management. Increasing the dose of C/F resulted in cross-linking of the sludge structure and increasing sludge density, which favored the incorporation of MP. The highest amount of PIX (5 mL/L), although most efficient for MP removal, resulted in 94 and 73% efficiencies of P-PO_4_ and COD removal, respectively, which may adversely affect the functioning of the biological part of the wastewater treatment plant.

## Figures and Tables

**Table 1:** Properties of the C/Fs.

Name of C/Fs (Company)	Chemical Composition	The Concentration of C/Fs Used (mL of Solution/L of Wastewater)
FPM (Korona JV)	Hydrocarbons: C_12_–C_15_, n-alkanes, isoalkanes, cyclic, <2% aromatic; ethoxylatedisotridecanol	1.10 mg/L
PEL (Stockhausen GMbH & Co.)	The copolymer of acrylamide and a cationic acrylic acid derivative in isoparaffin hydrocarbons	1.03 mg/L
FCT (Korona JV)	Adipic and sulfamide acid	0.90 mg/L
PIX (kemipol)	40–42% aqueous solution of iron(III) sulfate, consisting of 11.8 ± 0.4% of the total iron and up to 1% of free sulphuric acid	0.53 mg/L
PAX (kemipol)	Aqueous solution of polyaluminium chloride containing 17.0 ± 0.6% Al_2_O_3_ and 20.0 ± 2.0% of chloride ions	1.4 mg/L

## Data Availability

Not applicable.

## Supplementary Materials

The following supporting information can be downloaded at: https://www.mdpi.com/article/10.3390/ijerph192013013/s1, Figure S1: Photography of MP under UV light in the filter after filtration of wastewater
