# Supplementary figures and images for: Coagulation and Flocculation before Primary Clarification as Efficient Solutions for Low-Density Microplastic Removal from Wastewater
Source: Int J Environ Res Public Health. 2022 Oct 11;19(20):13013. doi: 10.3390/ijerph192013013 (PMC9602620; doi:10.3390/ijerph192013013)

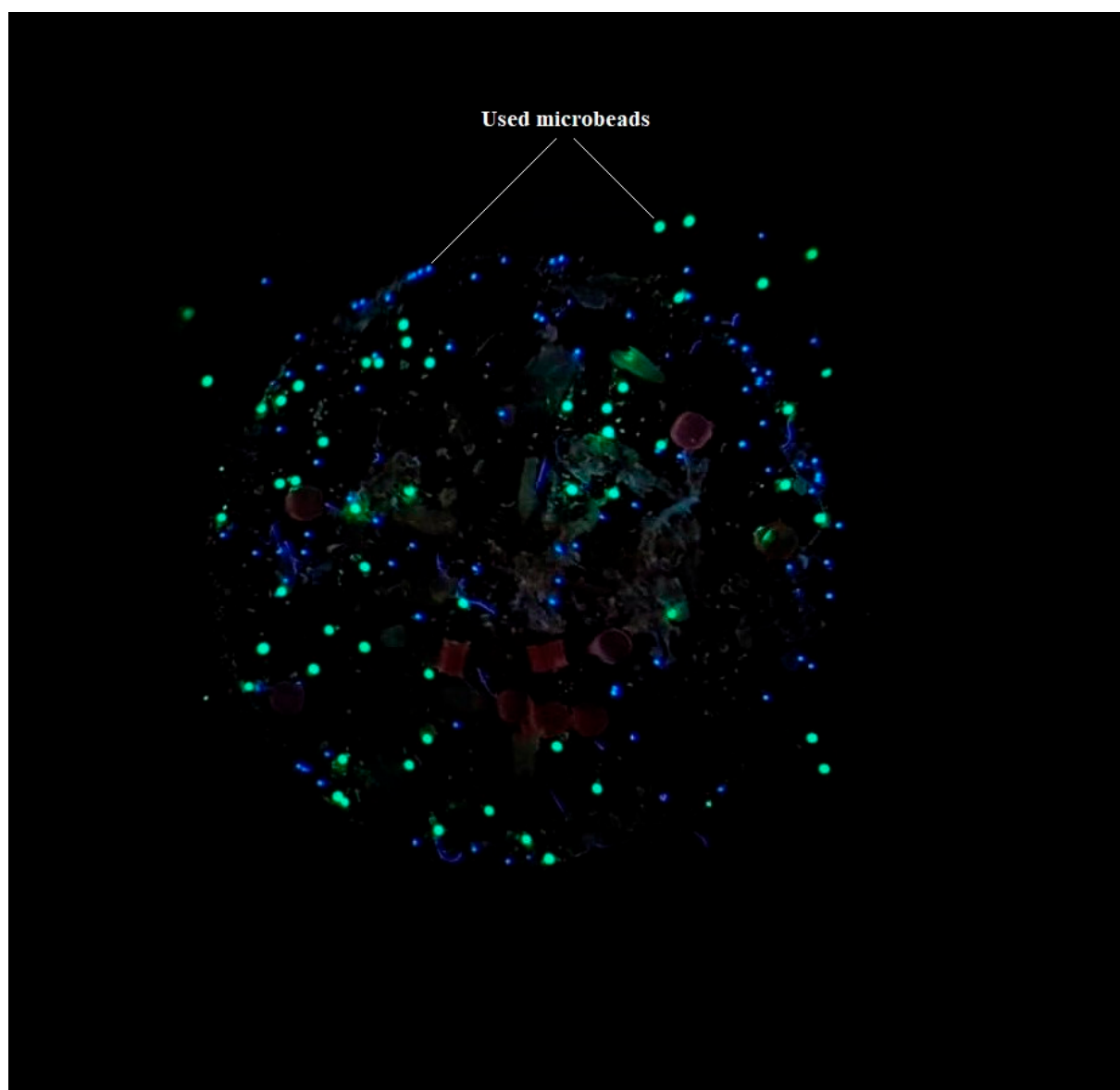

Figure S1: Photography of MP under UV light in the filter after filtration of wastewater

Supplement: Supplementary file 1 [file ijerph-19-13013-s001.zip › ijerph-1923672-supplementary.pdf]
